# Optically Induced Ferroelectric Polarization Switching in a Molecular Ferroelectric with Reversible Photoisomerization

**DOI:** 10.1002/advs.202102614

**Published:** 2021-10-29

**Authors:** Wei‐Qiang Liao, Bin‐Bin Deng, Zhong‐Xia Wang, Ting‐Ting Cheng, Yan‐Ting Hu, Shu‐Ping Cheng, Ren‐Gen Xiong

**Affiliations:** ^1^ Ordered Matter Science Research Center Nanchang University Nanchang 330031 P. R. China

**Keywords:** dielectric switching, molecular ferroelectrics, optical control, photoisomerization, polarization switching

## Abstract

Ferroelectrics usually exhibit temperature‐triggered structural changes, which play crucial roles in controlling their physical properties. However, although light is very striking as a non‐contact, non‐destructive, and remotely controlled external stimuli, ferroelectric crystals with light‐triggered structural changes are very rare, which holds promise for optical control of ferroelectric properties. Here, an organic molecular ferroelectric, *N*‐salicylidene‐2,3,4,5,6‐pentafluoroaniline (SA‐PFA), which shows light‐triggered structural change of reversible photoisomerization between *cis*‐enol and *trans*‐keto configuration is reported. SA‐PFA presents clear ferroelectricity with the saturate polarization of 0.84 *μ*C cm^−2^, larger than those of some typical organic ferroelectrics with thermodynamically structural changes. Benefit from the reversible photoisomerization, the dielectric real part of SA‐PFA can be reversibly switched by light. More strikingly, the photoisomerization enables SA‐PFA to show reversible optically induced ferroelectric polarization switching. Such intriguing behaviors make SPFA a potential candidate for application in next‐generation photo‐controlled ferroelectric devices. This work sheds light on further exploration of more excellent molecular ferroelectrics with light‐triggered structural changes for optical control of ferroelectric properties.

## Graphical abstract

Organic *N*‐salicylidene‐2,3,4,5,6‐pentafluoroaniline is a ferroelectric that experiences a light‐triggered structural change of reversible photoisomerization between *cis*‐enol and *trans*‐keto configuration, different from the general temperature‐triggered structural changes in other ferroelectrics. It has a saturate polarization of 0.84 *μ*C cm^−2^. The photoisomerization makes it show reversible optically induced ferroelectric polarization switching.

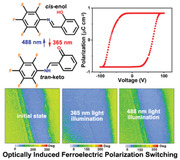

## 1 Introduction

Ferroelectrics are an important class of multifunctional materials with rich physical properties including ferroelectricity, dielectricity, and piezoelectricity, and wide technical applications such as in memory elements, capacitors, and sensors.^[^ ^1^ ^]^ Ferroelectric crystals usually experience temperature‐triggered structural changes,^[^ ^2^ ^]^ such as the displacement of Ti^4+^ ion in typcial ferroelectric BaTiO_3_ and the orientation change of [(CH_3_)_3_NCH_2_Cl]^+^ cation in [(CH_3_)_3_NCH_2_Cl]MnCl_3_.^[^ ^3^ ^]^ The temperature‐triggered structural change enables the manipulation of physical properties in ferroelectrics by temperature, like the switching of dielectric constant and the evolution of ferroelectric domain (a region with approximately uniform ferroelectric polarization).^[^ ^4^ ^]^ Since the first discovery of ferroelectricity in Rochelle salt in 1920,^[^ ^5^ ^]^ researchers have long and intensely studied ferroelectrics from the thermodynamic perspective.^[^ ^2^ ^]^ Besides typical temperature, other external stimuli, such as light, mechanical stress and magnetic field, also can induce structural changes.^[^ ^6^ ^]^ Among them, photoirradiation is very striking as a non‐contact, non‐destructive and remotely induced means,^[^ ^7^ ^]^ which motivates the relentless pursuit on optical control of ferroelectric properties.

Progress has been made in the light‐controllable ferroelectric polarization in some inorganic ferroelectrics.^[^ ^8^, ^9^, ^10^, ^11^, ^12^ ^]^ For example, high intensity laser illumination induced thermal effect results in the irreversible domain switching in LiNbO_3_, accompanied by the thermally induced surface damage.^[^ ^9^ ^]^ By using the mediation of photovoltaic effect, reversible optical control of ferroelectric domains has been achieved in BiFeO_3_.^[^ ^10^ ^]^ Polarized light induced ferroelectric domain wall motion was observed in BaTiO_3_ by modifying the stress at the domain wall.^[^ ^11^ ^]^ Alexei Gruverman *et al*. reported the optically controlled domain switching in BaTiO_3_‐based ferroelectric heterostructures, resulted from the redistribution of photogenerated carriers and screening charges at the interface.^[^ ^12^ ^]^ However, under light illumination, these inorganic ferroelectrics have not shown light‐triggered structural changes. To date, studies of optically controlled ferroelectric polarization are dominated by inorganic ferroelectrics with thermodynamic structural changes.^[^ ^8^, ^9^, ^10^, ^11^, ^12^ ^]^ The relationship between ferroelectric properties and light‐triggered structural changes is rarely explored because of the scarcity of ferroelectrics with such structural changes.^[^ ^6^ ^]^

Besides inorganic ferroelectrics, there is an attractive ferroelectric family of molecular ferroelectrics, which have attracted great attention in recent years because of the excellent ferroelectric properties comparable to those of the inorganic counterparts and the advantages in mechanical flexibility, light weight, good biocompatibility, and easy and environment‐friendly processing.^[^ ^13^, ^14^, ^15^, ^16^ ^]^ Moreover, the abundant species and structural diversity of molecular‐based materials provide rich opportunities for constructing molecular ferroelectrics with fascinating functions. In organic compounds, structural isomerizations are closely associated with light radiation, which have been extensively studied in photochromic crystals.^[^ ^17^ ^]^ Photoisomerizations, such as *trans*‐*cis* isomerization and enol‐ketone isomerization, can induce a type of light‐triggered structural changes that differ from the thermodynamic ones.^[^ ^17^ ^]^ It can be expected that, the ferroelectric properties are able to be optically controlled by reversible structural photoisomerizations. However, although the photoisomerization of organic photochromic crystals like salicylideneaniline derivatives have been extensively studied since the discovery of photochromism as early as 1867,^[^ ^17^, ^18^, ^19^ ^]^ the photoisomeric organic ferroelectrics remain sparse since ferroelectric must crystallize in one of the 10 polar point groups: 1 (*C*_1_), 2 (*C*_2_), *m* (*C_s_*), *mm*2 (*C*_2_ *_v_*), 4 (*C*_4_), 4*mm* (*C*_4_ *_v_*), 3 (*C*_3_), 3*m* (*C*_3_ *_v_*), 6 (*C*_6_), and 6*mm* (*C*_6_ *_v_*).^[^ ^2a^ ^]^ It is highly desired and urgent to construct photoisomeric organic ferroelectrics to combine the ferroelectricity with photoisomerization for exploiting the potential of optical control of ferroelectric properties.

In this study, we first synthesized a salicylideneaniline derivative *N*‐salicylidene‐4‐tert‐butylaniline (SA‐TBA) with obvious photoisomerization, but its centrosymmetric crystal symmetry disallows the possibility of ferroelectricity, as found in many other photoisomeric salicylideneanilines.^[^ ^17^, ^18^ ^]^ We then constructed a polar salicylideneaniline derivative *N*‐salicylidene‐3‐monofluoroaniline (SA‐MFA), while it shows no obvious photoisomerism and no clear ferroelectricity. We finally obtained a salicylideneaniline‐derived ferroelectric *N*‐salicylidene‐2,3,4,5,6‐pentafluoroaniline (SA‐PFA) with both polar crystal symmetry and remarkable photoisomerism at room temperature. SA‐PFA shows a light‐triggered structural change of photoisomerization between *cis*‐enol and *trans*‐keto configuration and exhibits clear ferroelectricity. Benefit from the photoisomerization, the ferroelectric domain of SA‐PFA can be optically switched and the optical polarization switching is intrinsic and completely reversible, which holds promise for light‐controlled ferroelectric devices. This pioneering work provides a promising paradigm for developing molecular ferroelectrics with optically controlled ferroelectric properties.

## 2 Results and Discussion

**Figure 1:**
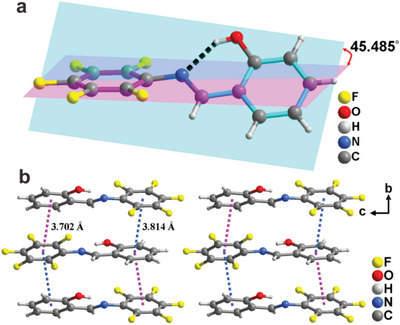
a) Asymmetric unit of crystal structure for SA‐PFA in *cis*‐enol form. The dash line denotes the O—H···N hydrogen bond. b) Packing view of crystal structure for SA‐PFA. The dash lines denote the *π*···*π* interactions.

We synthesized SA‐TBA, SA‐MFA, and SA‐PFA by the reaction of the salicylaldehyde and the corresponding anilines, as shown in the synthesis route for SA‐PFA (Figure S1, Supporting Information). Recrystallizing the as‐synthesized samples obtains the single crystals. The three compounds belong to the salicylideneaniline derived Schiff bases.^[^ ^17a^ ^]^ Schiff bases, named after Hugo Schiff, are nitrogen analogues of aldehydes or ketones in which the carbonyl groups have been replaced by imine or azomethine groups,^[^ ^20^ ^]^ which have the general formula of R_1_N = CR_2_R_3_, where R_1_ and R_2_ are aryl or alkyl groups, and R_3_ is aryl group, alkyl group, or hydrogen (Figure S2a, Supporting Information). For the R_1_N = CR_2_R_3_ formula of salicylideneaniline Schiff bases, the R_1_, R_2_, and R_3_ is the aryl group, 2‐hydroxy‐phenyl group, and hydrogen, respectively (Figure S2b, Supporting Information). In the case of SA‐TBA, SA‐MFA and SA‐PFA, the R_1_ is the 4‐tert‐butylphenyl, 3‐monofluorophenyl, and 2,3,4,5,6‐pentafluorophenyl group, respectively. The crystal structure determination reveals that SA‐TBA, SA‐MFA, and SA‐PFA crystallizes in the monoclinic centrosymmetric space group *P*2_1_/*n* (point group *C*_2_ *_h_*), orthorhombic polar space group *Pca*2_1_ (point group *C*_2_ *_v_*), and the monoclinic polar space group *P*2_1_ (point group *C*_2_) respectively, at room temperature (Table S1, Supporting Information). The absence of second harmonic generation (SHG) signal in SA‐TBA and the obvious SHG response observed in SA‐MFA and SA‐PFA further confirm the centrosymmetric crystal symmetry of SA‐TBA and the polar crystal symmetry of SA‐MFA and SA‐PFA, respectively (Figure S3, Supporting Information). Although SA‐TBA was found to show photoisomerization (Figure S4, Supporting Information),^[^ ^18a^ ^]^ its centrosymmetric crystal symmetry excludes the possibility to be a room‐temperature ferroelectric. Whereas both SA‐MFA and SA‐PFA satisfy the requirement of polar crystal symmetry for ferroelectrics, we thus focus on SA‐MFA and SA‐PFA. Structurally, their asymmetric units adopt an *cis*‐enol molecular configuration, which is the most stable form of salicylideneaniline derived Schiff bases. In the molecular structure, strong intramolecular O—H···N hydrogen bonds formed between the N atom of the N═C Schiff base and the H atom of the —OH of 2‐hydroxy‐phenyl group can be observed, with the O···N distance being of 2.610 Å for SA‐MFA and 2.624 Å for SA‐PFA (Figure S5a, Supporting Information, and **Figure**1a). The dihedral angle between two benzene rings from the salicylidene and the fluoroaniline is ≈6.925° in the SA‐MFA molecule, while in the SA‐PFA molecule, the dihedral angle is up to ≈45.485°, significantly larger than that in SA‐MFA molecule (Figure S5a, Supporting Information, and Figure **1**a). From the packing view of SA‐PFA, each polar SA‐PFA molecule is connected to the adjacent one through weak *π*···*π* interactions between two benzene rings from adjacent molecules, resulting in a stacked arrangement along the *b*‐axis (Figure 1b). Such an arrangement is beneficial for the generation of polarization in SA‐PFA with the crystal symmetry of *P*2_1_ space group, of which the orientation of spontaneous polarization is along the crystallographic *b*‐axis.

**Figure 2:**
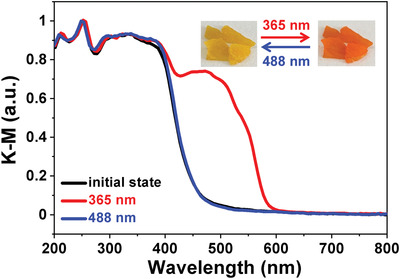
Photographs of polycrystals and solid‐state UV–vis spectra of SA‐PFA before and after 365 and 488 nm light irradiation at room temperature. The UV–vis spectra were transformed from the diffuse reflectance data by Kubelka‐Munk (K‐M) equation.

Differential scanning calorimetry (DSC) measurements reveal that SA‐MFA and SA‐PFA show no phase transition below melting point (Figures S5c and S6, Supporting Information), indicating no temperature‐triggered structural change. We then investigated their light‐triggered isomerizations. Photoisomerization of organic compounds are generally along with photochromism. We thus recorded the photographs of polycrystals and solid‐state UV–vis (ultraviolet‐visible) spectra before and after light irradiation to investigate the photochromic behavior of SA‐MFA and SA‐PFA. SA‐MFA shows no obvious changes in crystal color and absorption band before and after light irradiation (Figure S5d, Supporting Information), indicating that SA‐MFA is nonphotochromic. However, notable reversible photochromism was observed in SA‐PFA, of which the yellow polycrystals change into the orange‐red ones under irradiation of 365 nm UV (ultraviolet) light and return to the yellow ones after 488 nm visible light irradiation (**Figure** **2**). The UV–vis spectra of SA‐PFA also clearly show its photochromism (Figure 2). Before UV light irradiation, SA‐PFA absorbs light less than 450 nm, in good accordance with their yellow appearance. While after 365 nm UV light, a new absorption band emerges with the absorption edge up to ≈580 nm, which is consistent with the orange–red color. Although the light irradiation will induce the increase of temperature on the sample surface, the no obvious change of UV–vis spectra recorded at different temperatures, with the temperature much higher than the sample surface temperature after 365 nm light irradiation, indicates that the photochromism in SA‐PFA is not affected by the light‐irradiation induced photothermal effect (Figures S7 and S8, Supporting Information). Further irradiation with 488 nm visible light, this new absorption band disappears and the absorption spectrum returns to the initial state. The reversible photochromism of SA‐PFA is attributed to the reversible structural photoisomerization, while the small dihedral angle between two benzene rings in SA‐MFA restricts the generation of photoisomerization to make it show photochromism, as found in other salicylideneaniline derivatives.^[^ ^18^ ^]^ SA‐TBA also show reversible photochromism because of the large dihedral angle (48.679°) between two benzene rings for photoisomerization (Figure S4, Supporting Information). It is noted that the photochromism to the orange–red photoproducts (the *trans*‐keto form of SA‐PFA, see below) in SA‐PFA only happens at the crystal surfaces and not in the core of the crystals since the light can hardly penetrate into the bulk of the crystals, which was also found in other photochromic salicylideneaniline derivatives.^[^ ^21^ ^]^ We then examined the lifetime of the photoinduced *trans*‐keto form of SA‐PFA (Figures S9–S11, Supporting Information). When keeping the 365 nm light illuminated sample in dark, the intensity of the aforementioned new absorption band belonging to the photoinduced *trans*‐keto form of SA‐PFA shows a slow decrease with time and reduces to half of the initial intensity after 60 h (Figure S10, Supporting Information). Whereas, when keeping the illuminated sample under the 488 nm visible light irradiation for 10 s, the absorption band belonging to the photoinduced trans‐keto form of SA‐PFA disappears (Figure S11, Supporting Information). This indicates that the lifetime of photoinduced *trans*‐keto form of SA‐PFA in dark is much longer than that under visible light irradiation due to that the visible light irradiation will cause the back‐photoisomerization from *trans*‐keto form to the initial *cis*‐enol form. The lifetime of the photoinduced *trans*‐keto form of SA‐PFA in dark and under visible light irradiation is comparable to that found in other photochromic salicylideneanilines.^[^ ^22^ ^]^

**Figure 3:**
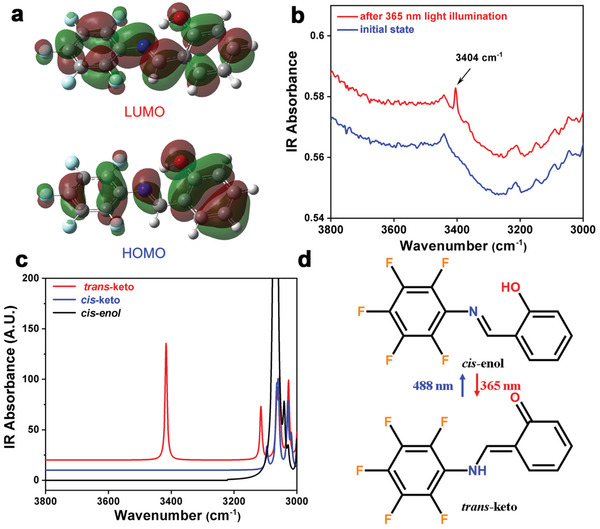
a) HOMO and LUMO of SA‐PFA. b) Experimental IR absorption spectra of SA‐PFA under ambient and after UV radiation of 365 nm. The newly emerging 3404 cm^−1^ peak is due to the N—H stretching vibration. c) Calculated IR absorption spectra of SA‐PFA with *cis*‐enol, *cis*‐keto, and *trans*‐keto forms, respectively. d) Scheme diagram showing the reversible structural photoisomerization of SA‐PFA between *cis*‐enol form and *trans*‐keto form.

We have tried to determine the crystal structure of SA‐PFA after photoisomerization, but we failed because the conversion rate of the photoisomerization for salicylideneaniline derivatives has been found to be too small for X‐ray structure determination.^[^ ^17^, ^18^ ^]^ We then deeply investigated the photoisomerization of SA‐PFA by other measurements and calculations. We first calculated the highest occupied molecular orbital (HOMO) and lowest unoccupied molecular orbital (LUMO) of cis‐enol SA‐PFA molecule. From the HOMO, the electron density is mainly distributed on the benzene ring (**Figure** **3**a). Compared with the HOMO, the LUMO has a significant shift to the C—N direction. The resultant energy gap between HOMO and LUMO is 0.1417 Hartree, corresponding to the energy of 3.86 eV, which is approximately consistent with the experimental UV–vis spectra. We then calculated the UV–vis spectra of SA‐PFA with *cis*‐enol, *cis*‐keto, and *trans*‐keto molecular configurations, respectively (Figure S12, Supporting Information). Compared with the enol form, the keto form displays new absorption bands at longer wavelengths. The new absorption peaks thus originate from the enol‐to‐keto conformational change of the SA‐PFA molecule after UV illumination.

Since the SA‐PFA molecule has both *cis*‐keto and *trans*‐keto forms, we further employed the infrared (IR) spectra to study the enol‐to‐keto photoisomerization. The experimental IR spectra before and after 365 nm UV light illumination did not show a significant change in absorbance (Figure S13, Supporting Information), which indicates a low conversion rate. Whereas, a new absorption peak centered at ≈3404 cm^−1^ emerges after UV irradiation (Figure 3b). Based on the experimentally measured single crystal X‐ray diffraction structure, we constructed several different molecular conformations, *cis*‐enol, *cis*‐keto, and *trans*‐keto forms (Figure S14, Supporting Information). Further calculation of infrared spectra reflects that only *trans*‐keto form displays a peak at the range larger than 3100 cm^−1^ (Figure 3c). The calculated wavenumber (3417 cm^−1^) is attributed to the free N—H stretching vibration, which is close to the measured IR absorption peak (3404 cm^−1^). Due to the formation of N—H···O hydrogen bond, the *cis*‐keto form has no free N—H stretching vibration mode. The most likely conformational change of the SA‐PFA molecule after 365 nm UV light illumination is thus from *cis*‐enol to *trans*‐keto form. Therefore, the aforementioned results clearly show that SA‐PFA undergoes a reversible light‐triggered structural change of molecular conformation between the *cis*‐enol and *trans*‐keto forms (Figure 3d), which is rarely found in ferroelectric crystals.^[^ ^2^ ^]^

**Figure 4:**
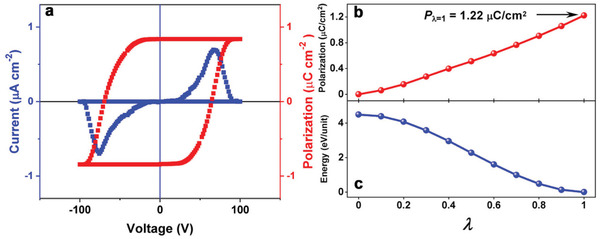
a) *P*−*E* hysteresis loops measured by the double‐wave method. b) Polarization and c) energy variation along the dynamic path connects the ferroelectric (*λ* = 1) and reference phase (*λ* = 0).

SA‐PFA adopts the polar crystal symmetry for ferroelectrics and exhibits a reversible light‐triggered structural change. We thus carried out the measurements of polarization–electric field (*P*–*E*) hysteresis loops to verify the ferroelectricity of SA‐PFA by the double‐wave method, which discriminates non‐hysteresis components from *P*–*E* hysteresis loops by applying identical unipolar waves twice and can compensate intricate non‐hysteresis components including electronic artifacts due to leakage in *P*–*E* loops.^[^ ^23^ ^]^ A typical *P*–*E* hysteresis loop with the saturate polarization (*P*_s_) being of 0.84 *μ*C cm^−2^ was obtained at room temperature on the thin film sample (**Figure** **4**a), clearly showing that SA‐PFA is a ferroelectric. Besides the double‐wave method with low frequency, we performed the positive up negative down (PUND) measurement with high frequency on SA‐PFA as well, while the poling time of PUND test is not long enough to switch the polarization (Figure S15, Supporting Information), which may be attributed to the relatively slow ferroelectric polarization switching in SA‐PFA. We also tried to measure the *P*–*E* hysteresis loop of SA‐MFA, which has a polar crystal symmetry and undergoes no light‐triggered structural change, but we failed to obtain the *P*–*E* hysteresis loop to prove its ferroelectricity. The *P*_s_ of SA‐PFA is larger than those of a number of organic ferroelectrics showing temperature‐triggered structural changes, such as some single‐component ones like TCAA (trichloroacetamide, 0.2 *µ*C cm^−2^),^[^ ^2b^ ^]^ DNP (1,6‐bis(2,4‐dinitrophenoxy)‐2,4‐hexadiyne, 0.24 *µ*C cm^−2^),^[^ ^2b^ ^]^ and TEMPO (2,2,6,6‐tetramethyl‐1‐piperidinyloxy, 0.5 *µ*C cm^−2^),^[^ ^2b^ ^]^ the organic salt (*R*)‐(*N*,*N*‐dimethyl‐3‐fluoropyrrolidinium) iodide (0.48 *µ*C cm^−2^),^[^ ^24^ ^]^ and some organic host–guest inclusions like [(2,6‐diisopropylanilinium)([18]crown‐6)]BF_4_ (0.3 *µ*C cm^−2^),^[^ ^25^ ^]^ [(2,6‐diisopropylanilinium)([18]crown‐6)]ClO_4_ (0.35 *µ*C cm^−2^),^[^ ^26^ ^]^ and [(4‐methoxyanilinium)([18]crown‐6)]BF_4_ (0.54 *µ*C cm^−2^),^[^ ^27^ ^]^ and is comparable to that of the organic co‐crystal Phz‐H_2_ba (0.8 *µ*C cm^−2^).^[^ ^2b^ ^]^

In order to gain deep insight into the ferroelectric polarization of SA‐PFA, density functional theory (DFT) calculation was carried out to evaluate the origin of polarization.^[^ ^28^ ^]^ According to the modern theory of polarization,^[^ ^29^ ^]^ the necessity of constructing polarization change path lies in selecting polarization quantum properly to avoid the wrong estimation of polarization value. The polarization switching process was calculated based on structure evolution along with the variation of structural parameters *λ*. A dynamic path between ferroelectric and reference states are constructed based on the crystal structure obtained from the single crystal X‐ray diffraction, while the other states are obtained from the matrix transformation of the coordinates considering the rotation of the C—N bond connecting the benzene ring (Figure S16, Supporting Information). As shown in Figure S16, Supporting Information, *λ* = 1, *λ* = 0, and *λ* = 0.9–0.1 represents the formal ferroelectric state with saturated polarization, the reference state with zero polarization, and the intermediate states connecting the ferroelectric and reference states, respectively. The variation of polarization as a function of the dynamic path is shown in Figure 4b, from which the polarization with 1.22 *μ*C cm^−2^ along *b*‐axis can be extracted from the ferroelectric configurations (*λ* = 1). While the polarization along *a*‐ and *c*‐axis is zero, which is in good accordance with the symmetry of space group *P*2_1_. The calculated ferroelectric polarization is closed to the experimental one. Along with the constructed path (0 < *λ* < 1), the polarization value changes smoothly, and turns to zero at *λ* = 0, which indicates a reference phase with zero polarization. At *λ* = 0, the molecule transforms into a planar conformation, which is perpendicular to the crystallographic *b*‐axis, so that the polarization along *b*‐axis becomes zero. At the same time, the reference state is a state with a maximum value of energy (Figure 4c). The energy barrier between the reference and the ferroelectric state represents the energy required for the internal rotation of SA‐PFA molecules. Therefore, the polarization switching is achieved by the rotation of two benzene rings in the molecule. The dipole moment of *cis*‐enol and *trans*‐keto forms are also calculated to investigate the polarization changes before and after light irradiation (Figure S17, Supporting Information). At ambient condition, SA‐PFA molecules adopt *cis*‐enol form with 2.70 Debye, whose dipole direction is approximately along N═C bond. After light illumination with 365 nm, the molecular dipole moment increased to 4.18 Debye as the molecule transforms to *trans*‐keto form. More importantly, the direction of the molecular dipole has changed dramatically. The dipole direction of *trans*‐keto form is nearly perpendicular to the C—N single bond (Figure S14c, Supporting Information). Further illumination with 488 nm can transfer the *trans*‐keto form back to *cis*‐enol form (Figure S17, Supporting Information). Therefore, the *cis*‐enol and *trans*‐keto configurations represent different polarization states with distinct different polarization directions.

**Figure 5:**
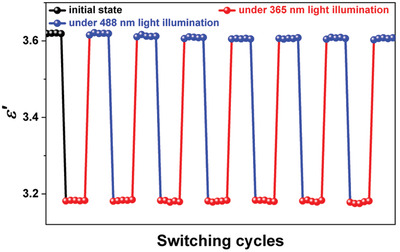
The real part (*ε*′) of the dielectric constant of SA‐PFA at 500 Hz before and after several cycles of 365 and 488 nm light irradiation at room temperature.

The aforementioned results indicate that SA‐PFA is a ferroelectric showing light‐triggered structural change of photoisomerization. We then investigated the effect of light on the dielectricity of SA‐PFA. The values of dielectric real part (*ε*′) and dielectric loss of SA‐PFA at different frequencies show no obvious change as the temperature varies (Figure S18, Supporting Information), which agrees with the fact that SA‐PFA undergoes no temperature‐induced structural change. When illuminating with 365 nm UV light, the *ε*′ presents a small decrease from initial value of ≈3.61 to ≈3.18, then the *ε*′ value recovers to the initial value after illumination with 488 nm visible light, showing the optically induced switching of dielectric real part (**Figure** **5**). This is consistent with the reversible light‐triggered structural change in SA‐PFA. The small optically induced change of dielectric constant in SA‐PFA may be attributed to the low conversion rate of the photoisomerization for salicylideneaniline derivatives. The *ε*′ keeps the initial value after several cycles of alternating 365 nm UV and 488 nm visible light illumination, indicating a good reversibility (Figure 5). After the 365 nm light is turned off, the *ε*′ value can remain ≈3.18 in dark for 300 s due to the slow decay of the *trans*‐keto form (Figures S19a and S10, Supporting Information). The *ε*′ value also keeps ≈3.61 in dark after the 488 nm light is turned off because that SA‐PFA remains the stable *cis*‐enol form after stopping 488 nm irradiation (Figure S19b, Supporting Information). We also recorded the *ε*′ of SA‐MFA without photoisomerization before and under 365 nm light irradiation, which shows a tiny increase of *ε*′ value from 3.85 to 3.89 because of the photothermal effect (Figure S20, Supporting Information). The 365 nm light irradiation induced decrease in *ε*′ value of SA‐PFA can be explained by investigating the intrinsic structural changes in the photoisomerization process. Under the 365 nm light irradiation, the molecular configuration of SA‐PFA changes from the initial *cis*‐enol form to the *trans*‐keto form. During this photoisomerization, obviously, the —OH groups and the intramolecular O—H···N hydrogen bonds in the *cis*‐enol form disappear in the *trans*‐keto form, which results in the change of *ε*′ value. Meanwhile, the functional —OH groups and the intramolecular O—H···N hydrogen bonds with asymmetric distribution of charges in the *cis*‐enol form contribute to a higher *ε*′ value, as found in other hydrogen‐bonded organic compounds.^[^ ^30^ ^]^

**Figure 6:**
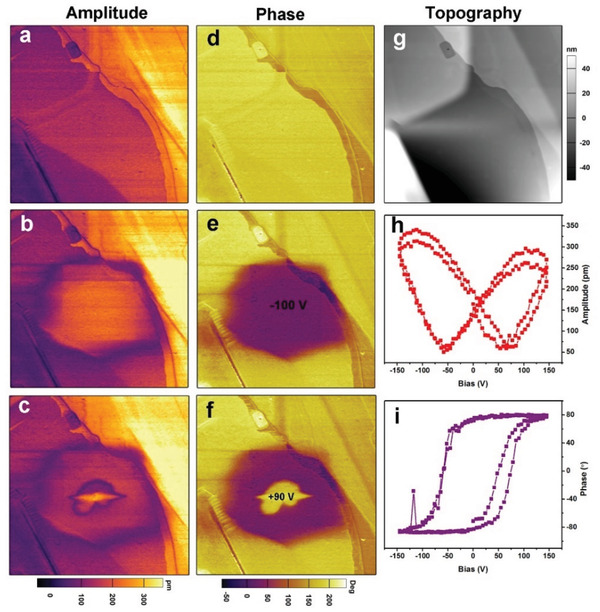
Electric field induced polarization switching behavior for the SA‐PFA. Out‐of‐plane PFM a–c) amplitude and d–f) phase images for a selected region in the thin film of SA‐PFA a,d) at the initial state, b,e) after poling with −100 V tip voltage over the central region and c,f) after the back switching operation, produced by poling the new domain with the tip voltage of +90 V. g) Topography image for the same region. h,i) Local PFM switching spectroscopy.

We further investigated the optically induced polarization switching in SA‐PFA. Before this investigation, we first studied the electric field induced polarization switching behavior by piezoresponse force microscopy (PFM) measurements. We conducted PFM tip poling experiments by applying voltage of −100 V on a selected area with initial single domain state (**Figure** **6**a,d), the domain switching process could be observed. The contrast of the out‐of‐plane PFM images are presented in Figure 6e, which correlated with the orientation of the out‐of‐plane component of the ferroelectric polarization, with the yellow domain pointing upward and purple domain pointing downward. Then, we demonstrate that the newly yield domain can be switched back. As shown in Figure 6c,f, one can see that partial purple domain switches to yellow. In addition, polarization reversal behavior of SA‐PFA crystal was demonstrated by measuring switching spectroscopy PFM (Figure 6h,i).

**Figure 7:**
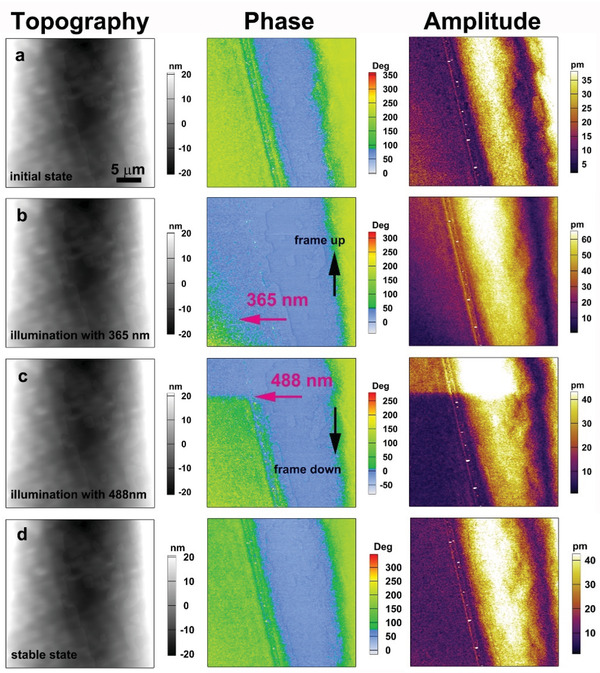
Optically induced polarization switching in SA‐PFA. PFM images acquired a) at initial state, b) after applying 365 nm UV light illumination, c) after applying 488 nm visible illumination from the moment mark by the red arrow and d) further irradiating with 488 nm visible light. The images from left to right are topography images, lateral PFM phase images and lateral PFM amplitude images.

The photoisomerization of SA‐PFA offers the possibility to optically control the polarization in it. **Figure** **7** shows the influence of 365 nm UV light and 488 nm visible light illuminations on the polarization of SA‐PFA. Figure 7a shows in‐plane PFM as well as simultaneously acquired topographic image at initial state in the dark condition. The acquired in‐plane PFM images show a stripe domain pattern. The out‐of‐plane PFM images for this region display a uniform response and points to single domain character of the out‐of‐plane component of the polarization (Figure S21a–c, Supporting Information). In order to monitor the role of the different light illumination on the domain structure in real time, the PFM imaging was performed continuously, as shown in Figure 7b–d. Figure 7b shows that the majority of yellow domain reversed to the blue domain after 365 UV illuminating. Note that, the full reversibility of the polarization induced by 365 UV light illumination can be achieved via a longer exposure time (Figure S22, Supporting Information). The isomerization times are the intrinsic properties of the SA‐PFA molecules. It has been reported to take place at picosecond to sub‐nanosecond timescales in some system.^[^ ^17b^ ^]^ The polarization did not completely reverse in a short time after 365 nm UV irradiation, which may be due to the need for domain walls in this region to balance other energies. The new domain structure modified by the 365 nm UV light illumination has no such consideration and thus could be switched instantaneous by the illumination of 488 nm visible light. As shown in Figure 7c where the scanning of the sample begins from the top of the image in the dark and then the 488 nm visible light is turned on at the moment indicated by a red arrow. One can see that the irradiation of the 488 nm visible light immediately leads to the change of the domain pattern. Further PFM imaging under the 488 nm visible light illumination is shown in Figure 7d. It shows that the domain structure has not changed further and is almost consistent with the one at the initial state. For the out‐of‐plane PFM, it shows a single domain state, consistent with the initial state as well (Figure S21d–f, Supporting Information). These results clearly show the optically induced polarization switching in SA‐PFA.

## 3 Conclusion

In summary, we have presented an organic photoisomeric ferroelectric, SA‐PFA, which experiences light‐induced structural change of reversible photoisomerization between *cis*‐enol and *trans*‐keto configuration. The saturate polarization of SA‐PFA reaches 0.84 *μ*C cm^−2^, comparable to that of organic ferroelectrics with thermodynamically structural changes. Owing to the photoisomerization, the dielectric real part of SA‐PFA can be reversibly switched by light. More importantly, the photoisomerization also enables SA‐PFA to show reversible optically induced ferroelectric polarization switching. These attributes make it great potential in applications for next‐generation photo‐controlled ferroelectric data storage and sensing. This work brings a new mechanism and material system for optical control of ferroelectric polarization. Considering the structural diversity and tunability of molecular‐based materials, more excellent molecular ferroelectrics with optically controlled ferroelectric properties can be expected.

## 4 Experimental Section

### Materials

All reagents and solvents in this experiment were of reagent grade and used without further purification.

For the synthesis of SA‐TBA, a mixture of 4‐(tert‐butyl)aniline (20 mmol) and salicylaldehyde (20 mmol) was refluxed in ethanol (50 mL) overnight. The crude product was obtained by removing the solvent by distillation under reduced pressure. The crystal of SA‐TBA was obtained by slowly evaporating the ethanol solution containing the crude product at room temperature.

For the synthesis of SA‐MFA, a mixture of 3‐fluoroaniline (20 mmol) and salicylaldehyde (20 mmol) was refluxed in ethanol (50 mL) for 12 h. The solvent was distilled under reduced pressure and a yellow crude product was obtained in good yield. The crystal of SA‐MFA was obtained by slowly evaporating the ethanol solution containing the crude product at room temperature.

For the synthesis of SA‐PFA, a mixture of 20 mmol 2,3,4,5,6‐pentafluoroaniline, 20 mmol salicylaldehyde, and 80 mL methanol was added into a 150 mL thick‐walled pressure flask and stirred at 353 K under airtight conditions for 10 h. The solution was distilled under reduced pressure and a yellow powder crude product was obtained after washing with n‐hexane. The crystal of SA‐PFA was obtained by slowly evaporating the acetone solution containing the crude product at room temperature. The phase purity of crystals of SA‐TBA, SA‐MFA, and SA‐PFA were verified by powder X‐ray diffraction (PXRD) (Figure S23, Supporting Information).

CCDC 2089814–2089816 contains the supplementary crystallographic data for this paper. These data can be obtained free of charge from The Cambridge Crystallographic Data Centre via www.ccdc.cam.ac.uk/data_request/cif.

### Thin‐Film Preparation

The precursor solution of SA‐PFA was prepared by dissolving 20 mg of crystals in 200 µL of acetone. Then, 20 µL of precursor solution was spread on a clean ITO (indium tin oxide)‐coated glass (1 × 1 cm^2^). The thin film was obtained after slowly evaporating the solution at room temperature for 20 min. The PXRD patterns of the thin‐film sample show (001) preferred orientation (Figure S24, Supporting Information), as reflected from the (001) and (002) reflections of SA‐PFA with strong intensity. Additionally, the PXRD patterns also clearly present other reflections of SA‐PFA thin film including (10‐1), (101), (111), (020), (120), and (221). The clear (020) reflection corresponds to the orientation along the polar *b*‐axis, making us obtain the *P*–*E* hysteresis loops and the domain switching on the thin‐film sample. It is noted that the low intensity of (020) reflection means a small fraction of the crystals in the film were oriented to the polar *b*‐axis, thus it was needed to try many times on selected area of the film to successfully obtain the *P*–*E* hysteresis loops.

### Measurements

For the single‐crystal X‐ray diffraction experiments, the data at 293 K was collected by using a Rigaku CCD diffractometer with Mo‐K*α* radiation (*λ* = 0.71073 Å). Data processing including empirical absorption correction, cell refinement, and data reduction was performed by using CrystalClear 1.3.5 (Rigaku). The crystal structures were solved by the direct method and refined the crystal structures by the full‐matrix method based on *F* ^2^ with the SHELXL2014 software package. The C, N, O, and F atoms were refined anisotropically. The positions of H atoms were generated geometrically.

For the SHG measurements, an unexpanded laser beam with low divergence (pulsed Nd:YAG at a wavelength of 1064 nm, 5 ns pulse duration, 1.6 MW peak power, 10 Hz repetition rate) was used. The instrument model is Ins 1210058, INSTEC Instruments, while the laser is Vibrant 355 II, OPOTEK. SHG experiments were performed on the polycrystalline powder sample, which was prepared by grinding the as‐grown crystals into fine powder.

For the DSC measurements, NETZSCH DSC 214 instrument was used. The polycrystalline powder samples (6.0, 7.8, and 13.1 mg for SA‐PFA, SA‐TBA, and SA‐MFA, respectively) were placed in aluminum crucibles, and measured under nitrogen atmosphere with a heating rate of 10 K min^−1^.

The UV–vis diffuse reflectance spectra were measured on a Shimadzu UV‐3600Plus spectrophotometer equipped with the integrating sphere (ISR‐603) with the wavelength range of 200–800 nm. BaSO_4_ was used as a 100% reflectance reference. The reflectance data was then converted to absorbance data by using the Kubelka–Munk (K–M) equation.^[^ ^31^ ^]^ UV–vis spectra of the polycrystalline powder sample was recorded before and after 365 (for two minutes) and 488 nm (for 10 s) light irradiation. The 365 nm LED light source and 488 nm LED light source was used to provide the required irradiation, of which the light intensity was ≈105 and 60 mW cm^−2^, respectively. For SA‐PFA, the UV–vis spectra after different 365 nm light illumination time and the change of UV–vis spectra with time after 365 nm light irradiation were also recorded.

The IR spectra were recorded on a Bruker INVENIO‐R spectrometer with KBr pellets at room temperature for the polycrystalline powder sample before and after 365 nm light irradiation (two minutes), which was realized by the 365 nm LED light source with the light intensity of ≈105 mW cm^−2^.

For the *P*–*E* hysteresis loop measurements, the double‐wave method was used, which was carried out on a home‐built system consisting of Keithley 6517B electrometer/high resistance meter, Keysight 33500B series waveform generator, and Keithley 4200A‐SCS parameter analyzer. The *P*–*E* hysteresis loop was measured on the thin film sample with thickness of ≈2.5 µm, which was fabricated by the solution method of spreading 20 µL precursor solution of SA‐PFA (20 mg as‐grown crystals in 200 µL acetone) on ITO/glass substrate. Thus, the down‐electrode material is ITO. The up‐electrode material is liquid gallium indium alloy (Ga 75.5%/In 24.5%, Sigma‐Aldrich), which can be transferred and deposited on thin film easily. The waveform of the applied voltage of double‐wave method is shown in Figure S25, Supporting Information. The complete test takes a total of 120 s and has a poling voltage of 100 V. The test has a pair of positive and negative preset poling waves to switch the polarization before the “double wave”. At the left and right ends of the preset voltage, a waiting time or delay period of 10 s, was set to release external charge and exclude some extrinsic electrical signals. Then a pair of “double wave” was applied for a period of 60 s. The PUND measurement was carried out by using the Ferroelectric Tester (Radiant Technologies, Inc.) at a pulse width of 1 ms and a drive voltage of 200 V.

The dielectric measurements were performed by using the Tonghui TH2828A impedance analyzer. For the dielectric measurements under or after light irradiation, polycrystalline sample (15 mg) of SA‐PFA was melted at 428 K and sandwiched by two ITO‐coated glasses (1.5 × 1.8 cm^2^), and then slowly cooled down the temperature to room temperature to first prepare the non‐illuminated film sample (thickness, 28.11 µm). The transparent ITO‐coated glasses were used as the electrodes (active area, ≈225 mm^2^), which allowed the light to pass through them. Optical illumination of the sample was realized by using 365 nm LED light source and 488 nm LED light source, of which the light intensity is ≈105 and 60 mW cm^−2^, respectively. The light incidence was normal to the sample surface with the light spot larger than the sample. The real part ( *ε*′) of the dielectric constants of SA‐PFA before light irradiation, under 365 nm light irradiation, and under 488 nm light irradiation were then recorded at 500 Hz at room temperature, which were performed in a dark room to avoid the influence of external light. For the measurements of temperature‐dependent and frequency‐dependent dielectric constant, the non‐illuminated sample of powder‐pressed round pellets (diameter, 13 mm; thickness, 0.55 mm) of SA‐PFA was used. Carbon conducting paste was painted fully on the large faces of the pellets as the electrodes (active area, ≈132.66 mm^2^). The temperature‐dependent *ε*′ and dielectric loss of SA‐PFA from 290 to 380 K at 10, 100, and 1000 kHz and the frequency‐dependent *ε*′ and dielectric loss of SA‐PFA from 100 Hz to 1000 kHz at room temperature were then recorded.

PFM measurements were conducted by a resonant‐enhanced PFM (Cypher ES, Asylum Research). Conductive Pt/Ir‐coated silicon probes (EFM‐50, Nanoworld) were used. PFM imaging was performed by applying an AC modulation bias with drive amplitude of 10 V peak near the contact resonance frequency that is in the range of 300–350 kHz for out‐of‐plane PFM mode and 620–660 kHz for the in‐plane PFM mode. The thin film sample of SA‐PFA with thickness of ≈2.5 µm was used for the PFM measurements, of which the fabrication is similar to that in the *P*–*E* hysteresis loop measurements. Optical illumination of the sample was realized by using a custom‐designed optical source integrated with the PFM system. The 365 and 488 nm LED light source was used to provide the required irradiation, of which the light intensity is ≈105 and 60 mW cm^−2^, respectively.

PXRD data was measured on the Rigaku Smartlab X‐ray diffractometer for both the polycrystalline sample and thin film sample, and the diffraction patterns were collected in the 2*θ* range of 5°–40° with a step size of 0.02°.

### Calculation Condition

The HOMO, LUMO, UV–vis, and IR are calculated at b3lyp/6‐31G(d) level with Gaussian 09 software. DFT calculations were carried out based on the Berry phase method developed by Kingsmith and Vanderbilt.^[^ ^32^ ^]^ The first‐principles calculations were performed within the framework of DFT implemented in the Vienna Ab initio Simulation Package.^[^ ^33^ ^]^ The energy cut‐off for the expansion of the wave functions was fixed to 550 eV and the exchange‐correlation interactions were treated within the generalized gradient approximation of the Perdew–Burke–Ernzerhof type.^[^ ^34^ ^]^ For the integrations over the k‐space a 4 × 3 × 2 k‐point mesh was used. The experimental room temperature (293 K) crystal structure was relaxed with unit cell parameters remain unchanged. Then the relaxed structure was used as the ground state for evaluating the ferroelectric polarization. The intramolecular rotation of the SA‐PFA was realized by matrix transformation of the coordinates using Microsoft Excel.

## Conflict of Interest

The authors declare no conflict of interest.

## Supporting information

Supporting Information

## Data Availability

Research data are not shared.
