# Supplementary material for: Optically Induced Ferroelectric Polarization Switching in a Molecular Ferroelectric with Reversible Photoisomerization
Source: Adv Sci (Weinh). 2021 Oct 29;8(24):2102614. doi: 10.1002/advs.202102614 (PMC8693059; doi:10.1002/advs.202102614)
Supplement: Supplementary file 1 — Supporting Information [file ADVS-8-2102614-s001.pdf]

ADVANCED SCIENCE
Open Access

Supporting Information

for *Adv. Sci.*, DOI: 10.1002/advs.202102614

Optically Induced Ferroelectric Polarization Switching in a Molecular Ferroelectric with Reversible Photoisomerization

Wei-Qiang Liao, Bin-Bin Deng, Zhong-Xia Wang*, Ting-Ting Cheng, Yan-Ting Hu, Shu-Ping Cheng, and Ren-Gen Xiong**

Supporting Information

Optically Induced Ferroelectric Polarization Switching in a Molecular Ferroelectric with Reversible Photoisomerization

Wei-Qiang Liao, Bin-Bin Deng, Zhong-Xia Wang*, Ting-Ting Cheng, Yan-Ting Hu, Shu-Ping Cheng, and Ren-Gen Xiong**

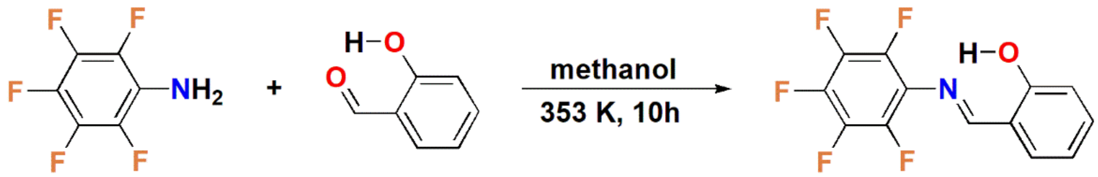

Figure S1. The synthesis route of SA-PFA.

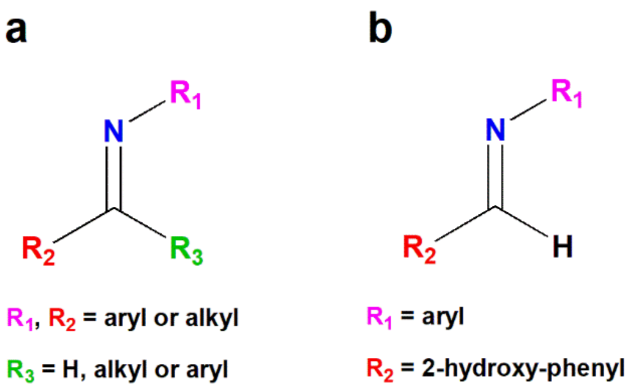

Figure S2. General molecular structures of a) Schiff bases and b) salicylideneaniline derived Schiff bases.

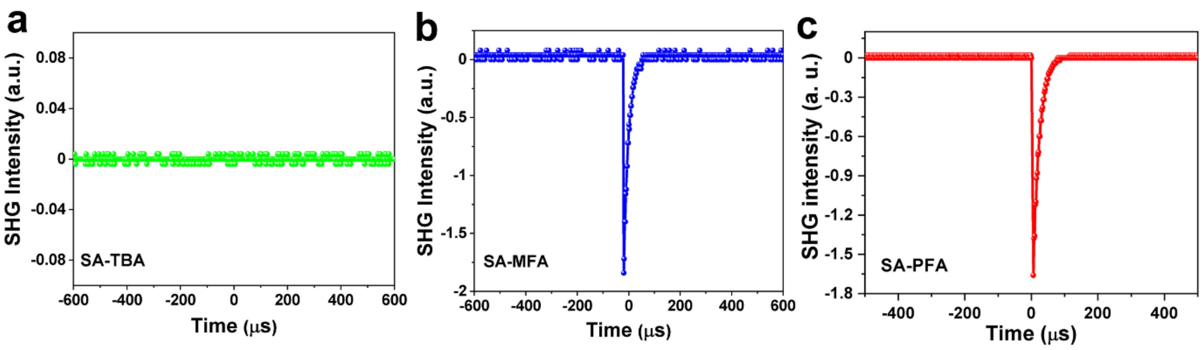

Figure S3. SHG signal of a) SA-TBA, b) SA-MFA, and c) SA-PFA at room temperature.

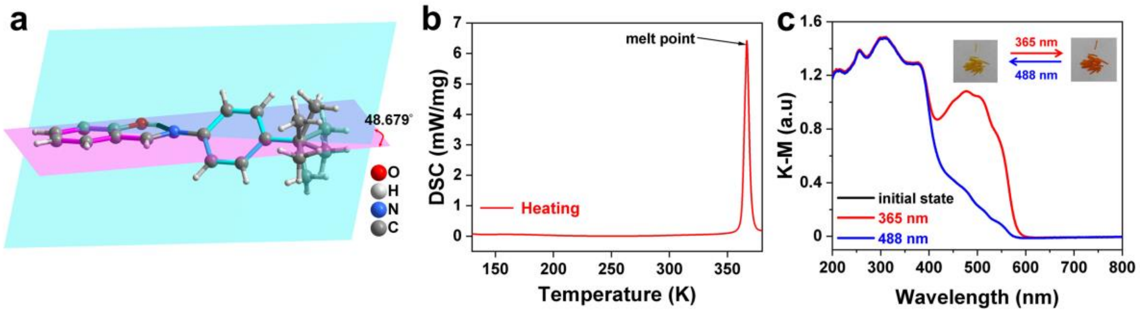

Figure S4. a) Asymmetric unit of crystal structure for SA-TBA in *cis*-enol form. b) DSC curves of SA-TBA. c) Photographs of polycrystals and solid-state UV-vis spectra of SA-TBA before and after light irradiation at room temperature, showing obvious photochromism. The UV-vis spectra were transformed from the diffuse reflectance data by Kubelka-Munk (K-M) equation.

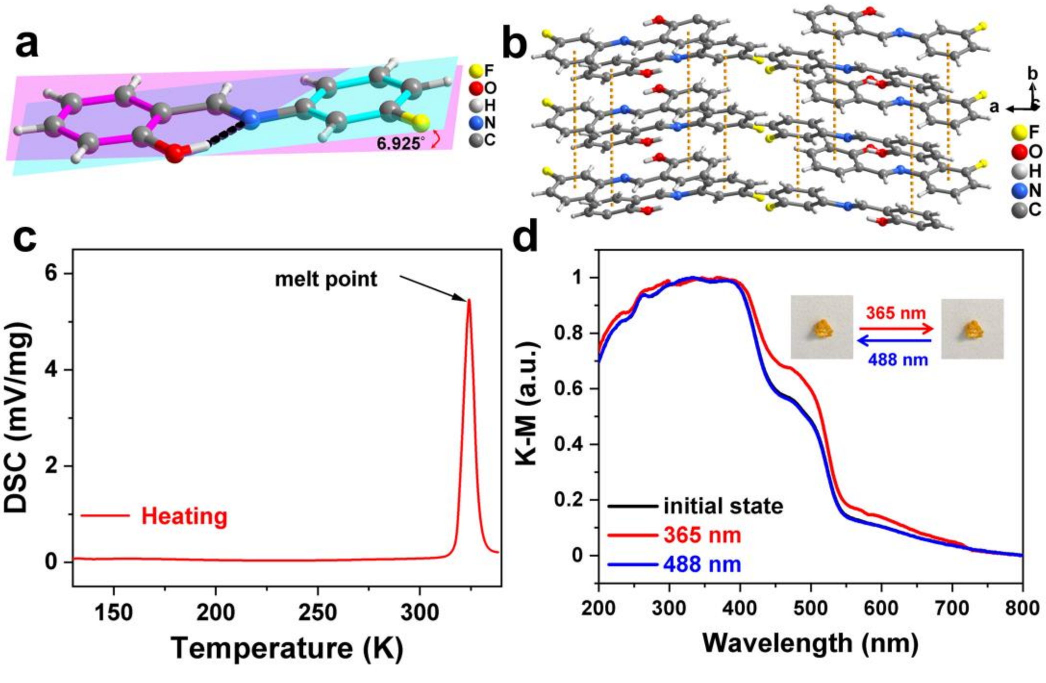

Figure S5. a) Asymmetric unit of crystal structure for SA-MFA in *cis*-enol form. The dash line denotes the O-H···N hydrogen bond. b) Packing view of crystal structure for SA-MFA. The dash lines denote the π···π interactions with average π···π distance of 3.842 Å. c) DSC curves of SA-MFA. d) Photographs of polycrystals and solid-state UV-vis spectra of SA-MFA before and after light irradiation at room temperature, showing no obvious

photochromism. The UV-vis spectra were transformed from the diffuse reflectance data by Kubelka-Munk (K-M) equation.

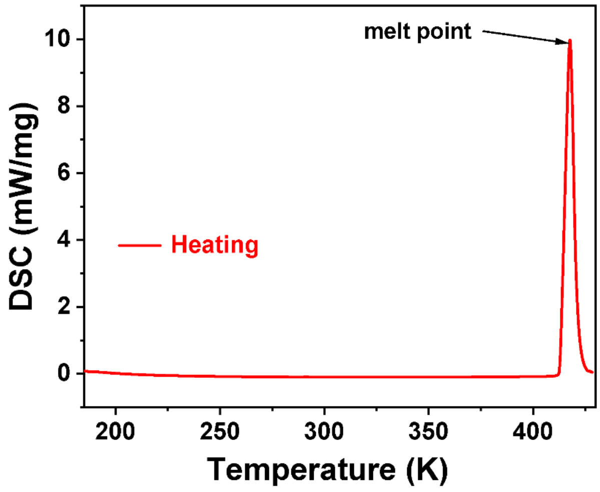

Figure S6. DSC curve of SA-PFA.

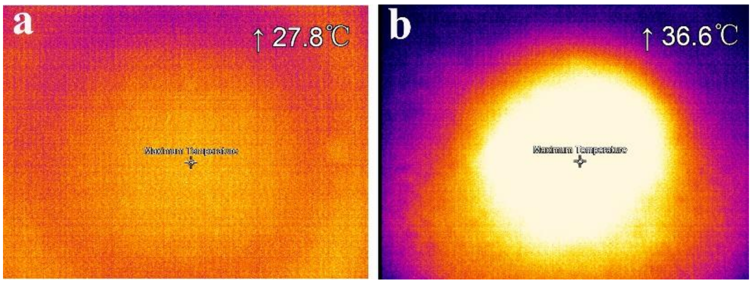

Figure S7. Change of surface temperature of SA-PFA polycrystalline sample after 365 nm light irradiation for 5 minutes, longer than the illumination time to trigger the photoisomerization, recorded by an infrared thermography camera.

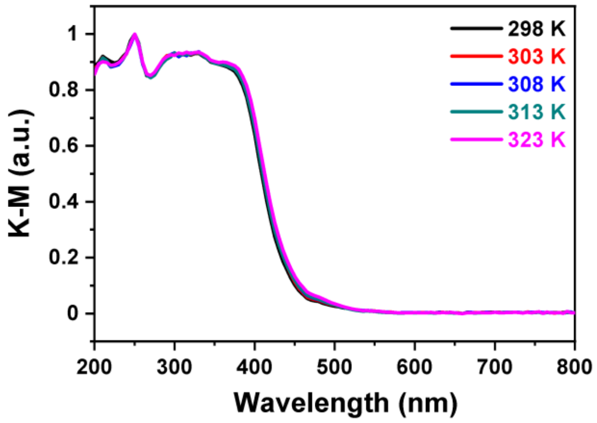

Figure S8. Solid-state UV-vis spectra of non-illuminated SA-PFA from 298 K to 323 K. The UV-vis spectra were transformed from the diffuse reflectance data by Kubelka-Munk (K-M) equation.

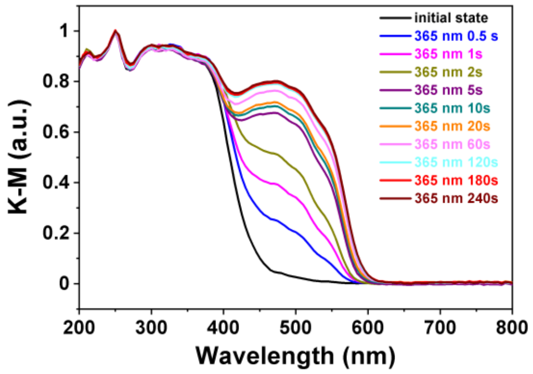

Figure S9. Solid-state UV-vis spectra of SA-PFA recorded before and after 365 nm light irradiation for different illumination time. The UV-vis spectra were transformed from the diffuse reflectance data by Kubelka-Munk (K-M) equation.

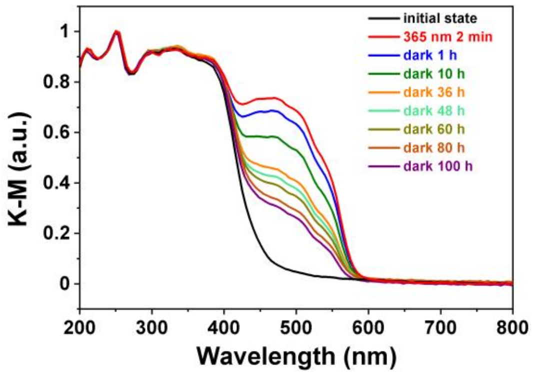

Figure S10. Change of solid-state UV-vis spectra of SA-PFA with time after stopping 365 nm light irradiation and keeping the illuminated sample in dark. The UV-vis spectra were transformed from the diffuse reflectance data by Kubelka-Munk (K-M) equation.

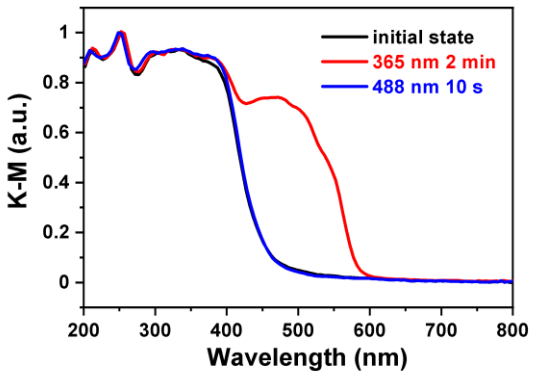

Figure S11. Change of solid-state UV-vis spectra of SA-PFA after stopping 365 nm light irradiation and keeping the illuminated sample under the 488 nm visible light irradiation. The UV-vis spectra were transformed from the diffuse reflectance data by Kubelka-Munk (K-M) equation.

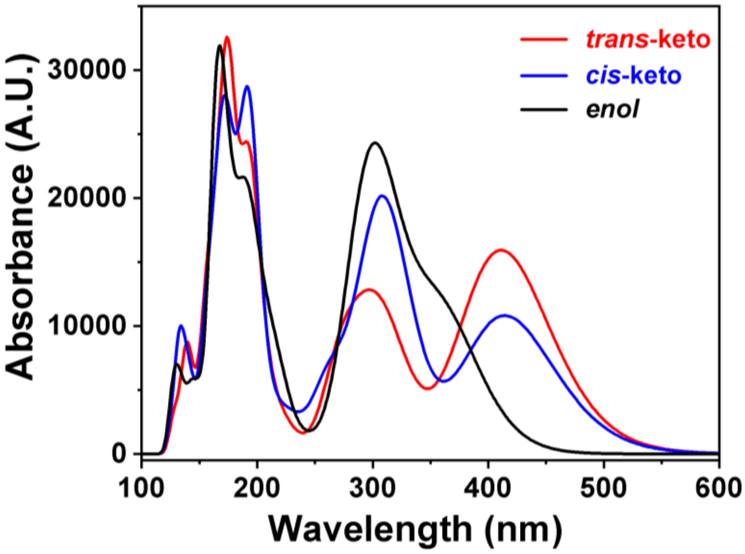

Figure S12. Calculated UV-vis absorption spectra of SA-PFA with *cis*-enol, *cis*-keto and *trans*-keto forms, respectively.

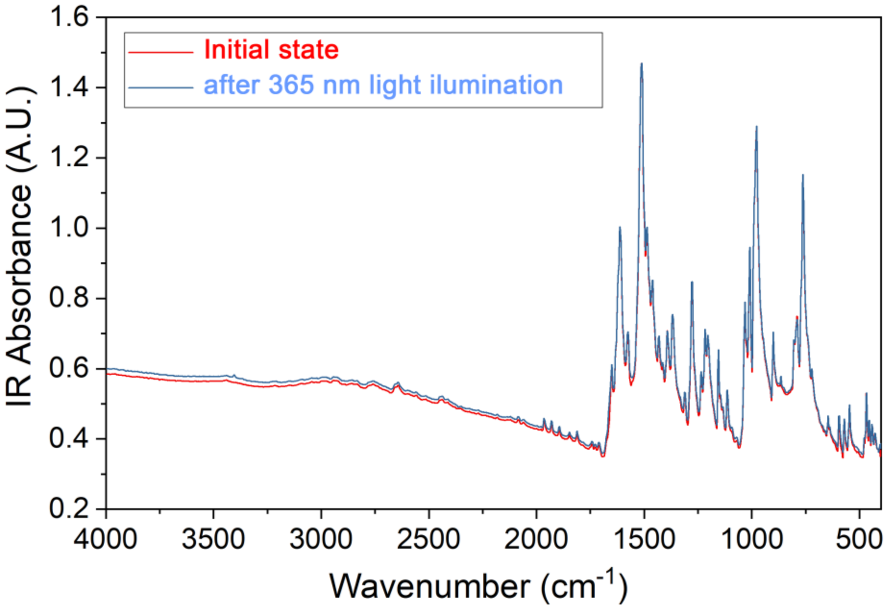

Figure S13. Experimental full range IR absorption spectra of SA-PFA at ambient and after UV radiation of 365 nm.

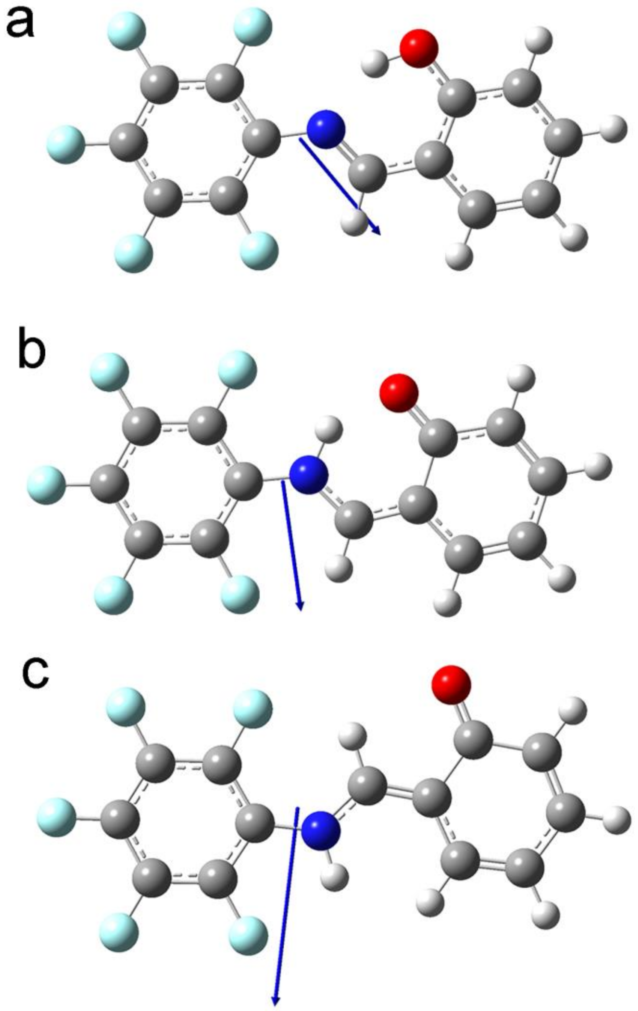

Figure S14. Molecular structure of SA-PFA with a) *cis*-enol form 2.70 Debye, b) *cis*-keto form 2.77 Debye and c) *trans*-keto form 4.18 Debye.

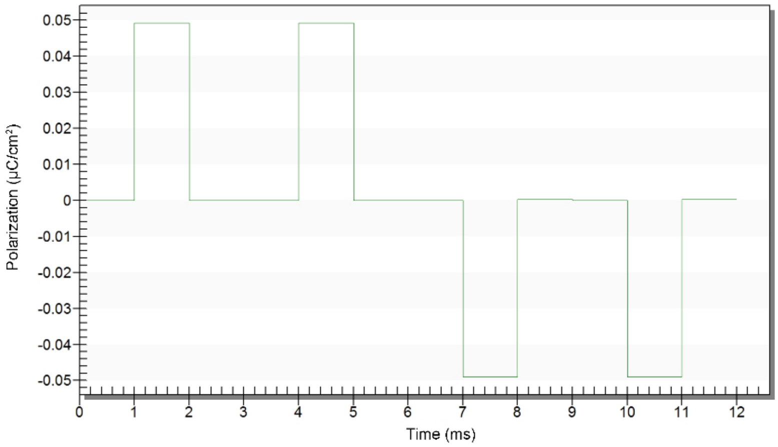

Figure S15. The result of PUND test for SA-PFA at a pulse width of 1 ms and a drive voltage of 200 V, showing a zero Δ*P* (*P* is the polarization).

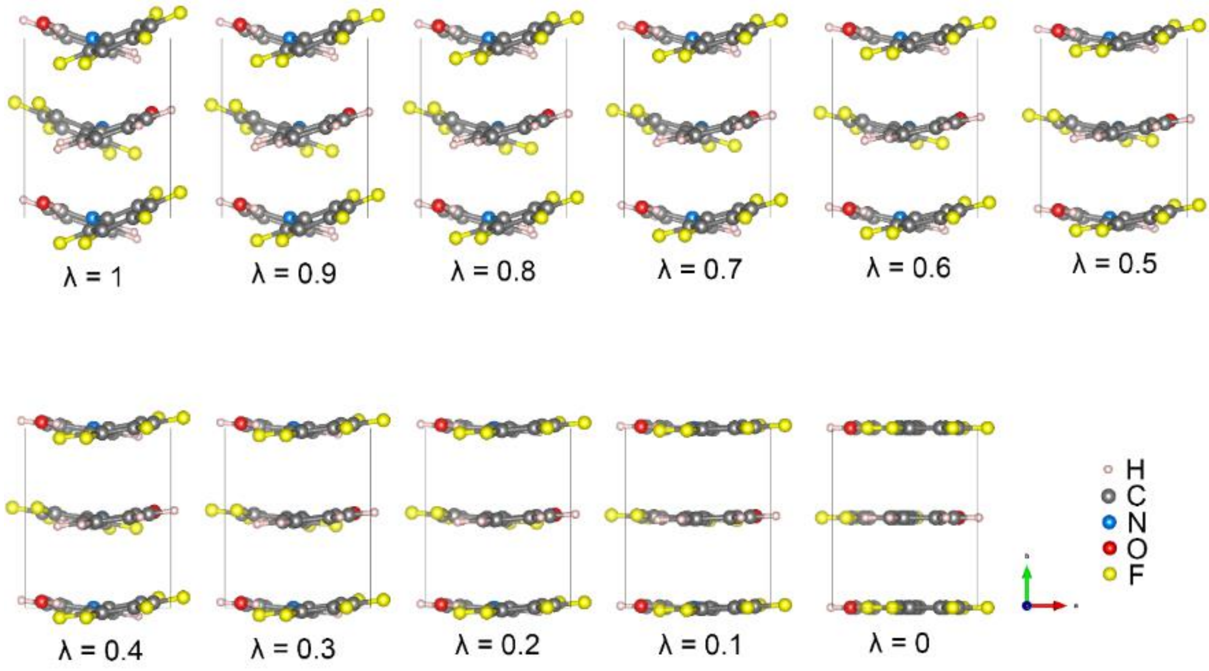

Figure S16. Structural evolution along with the structural parameters λ, the direction of ferroelectric polarization is along the crystallographic *b*-axis.

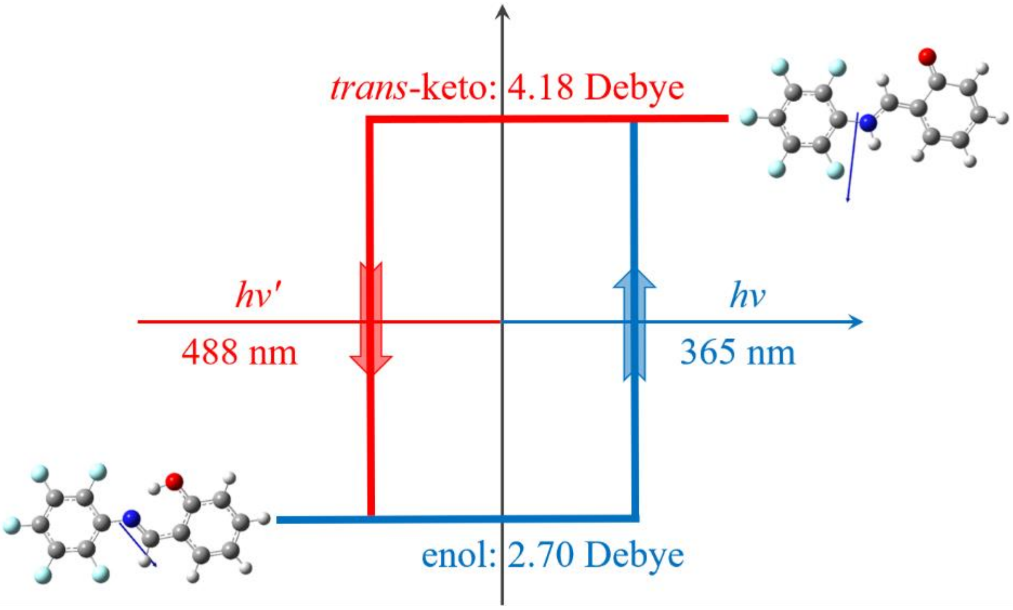

Figure S17. Schematic diagram of molecular conformation transformation of SA-PFA, the blue arrow in the molecule indicates the direction of the molecular dipole.

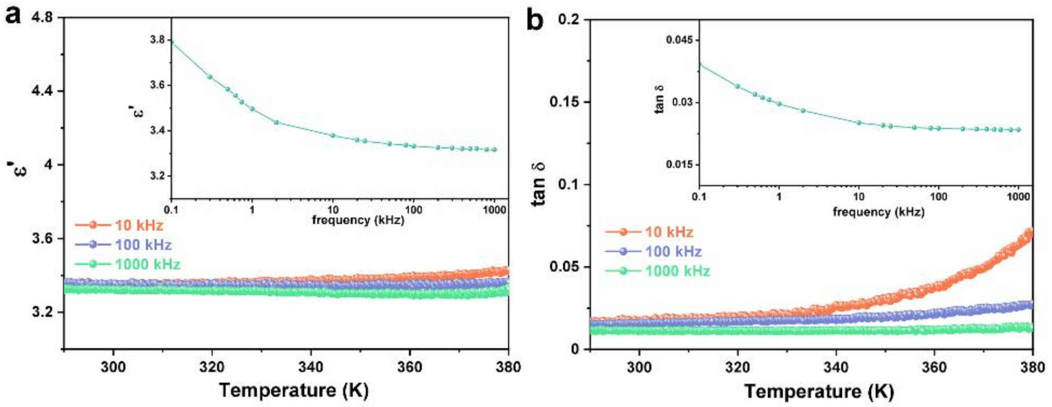

Figure S18. Temperature-dependent a) dielectric real part and b) dielectric loss of SA-PFA at different frequencies. Insets show the frequency-dependent dielectric real part and dielectric loss of SA-PFA at room temperature, presenting a decrease of value with the increase of frequency.

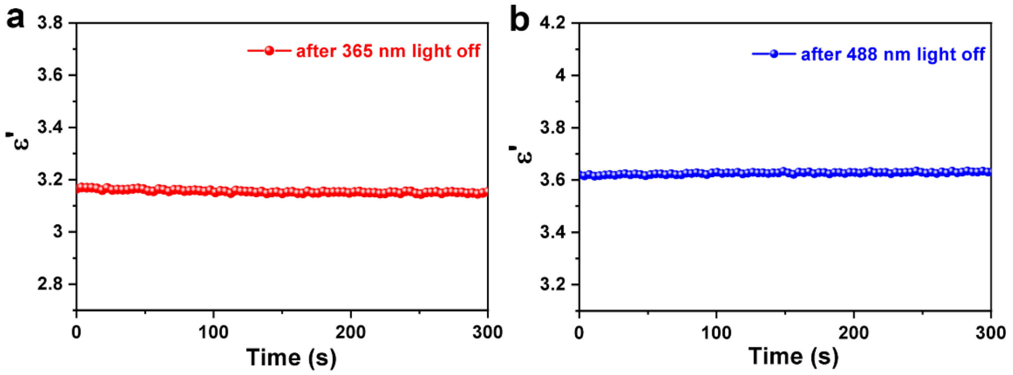

Figure S19. The real part (ε') of the dielectric constant of SA-PFA recorded after stopping 365 nm a) and 488 nm b) light irradiation in dark.

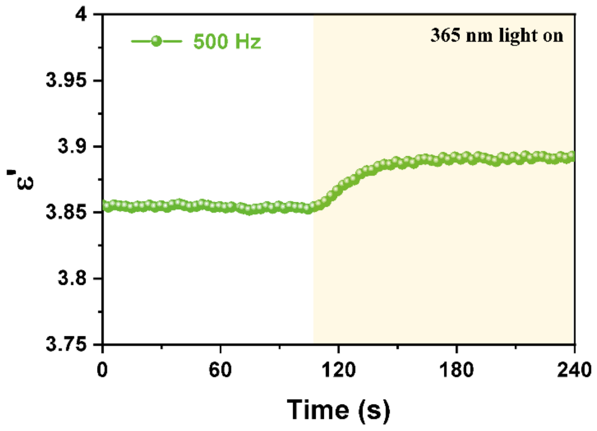

Figure S20. The real part (ε') of the dielectric constant of SA-MFA at 500 Hz before and under 365 nm light irradiation.

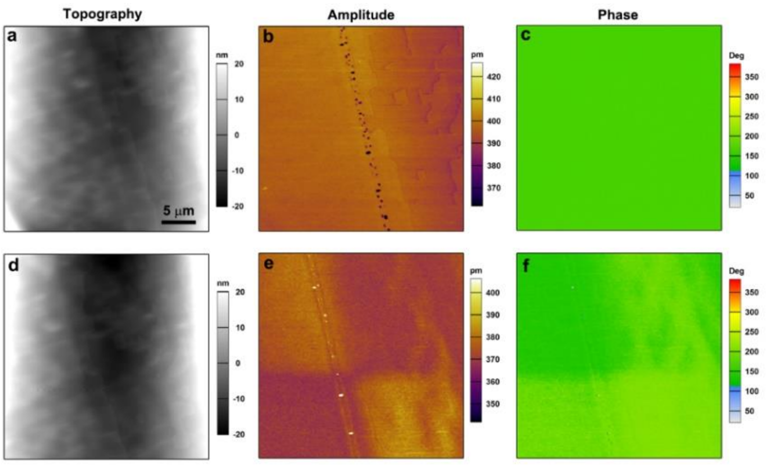

Figure S21. a-c) Out-of-plane PFM images for the region shown in Figure 7a at initial state. d-f) Out-of-plane PFM imaging under 488 nm light illumination after the in-plane PFM imaging in Figure 7d.

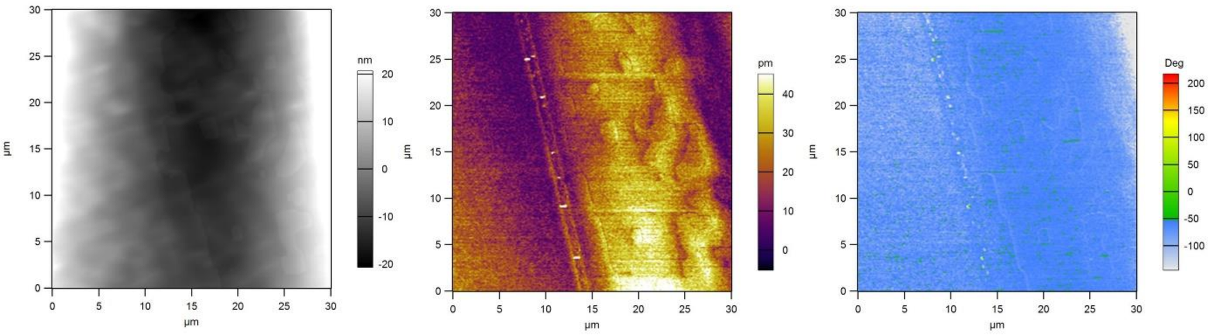

Figure S22. The post optical poling of the domain structure shown in Figure 7b, which was acquired after 45 min of continuous 365 nm UV light exposure, showing a single domain state.

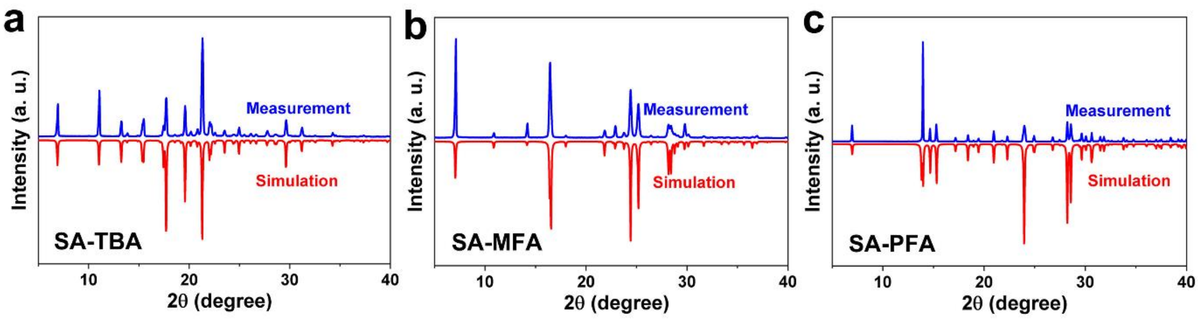

Figure S23. Experimental PXRD patterns of a) SA-TBA, b) SA-MFA, and c) SA-PFA at room temperature matching with the simulated ones from crystal structures.

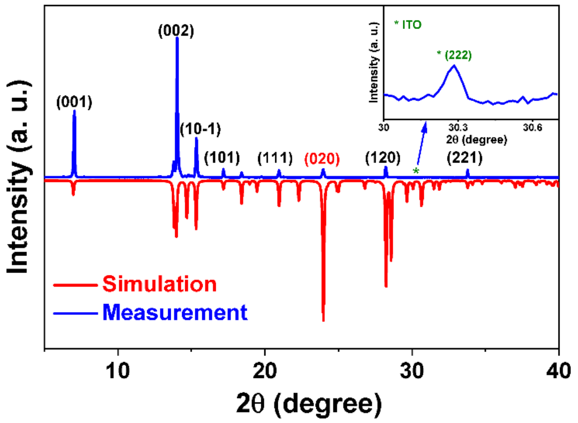

Figure S24. Experimental PXRD patterns of the thin film sample of SA-PFA grown on the ITO/glass substrate, clearly showing the (020) reflection. The background looks flat because of the very strong intensity of PXRD patterns of SA-PFA thin film. The PXRD patterns at around 30.3° is enlarged to show the (222) reflection of ITO.

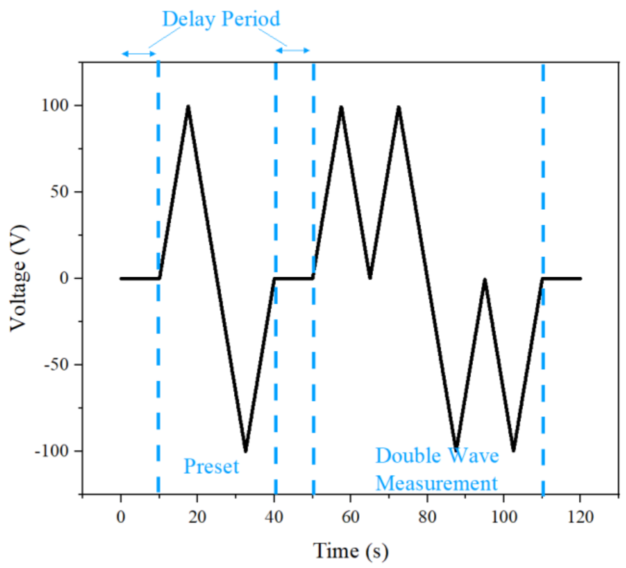

Figure S25. *Voltage–time* waveform of double-wave method in our measurements.

Table S1. Crystal data and structure refinement for SA-MFA, SA-PFA, and SA-TBA.

**Compound**	**SA-MFA**	**SA-PFA**	**SA-TBA**
Temperature	293 K	293 K	293 K
Formula	$C_{13}H_{10}FNO$	$C_{13}H_6F_5NO$	$C_{17}H_{19}NO$
Weight	215.22	287.19	253.33
Crystal system	orthorhombic	monoclinic	monoclinic
Space group	$Pca2_1$	$P2_1$	$P2_1/n$
a (Å)	25.005(4)	6.0921(2)	14.3393(3)
b (Å)	3.9210(7)	7.4199(2)	6.40790(10)
c (Å)	10.7140(18)	12.7941(4)	17.2050(3)
β (°)	90	98.421(3)	111.301(2)
V (Å^3)	1050.4(3)	572.09(3)	1472.88(5)
Z	4	2	4
R_{int}	0.0829	0.0331	0.0244
R_1	0.1127	0.1203	0.0719
wR_2	0.2868	0.2705	0.2211
GOF	1.242	1.415	1.079
